# HUMAn, a Real-Time Evolutive Patient Model for Major Incident Simulation: Development and Validation Study

**DOI:** 10.2196/66201

**Published:** 2025-03-07

**Authors:** Maxence Laurent, Arnaud Jaccard, Laurent Suppan, Elio Erriquez, Xavier Good, Eric Golay, Dominique Jaccard, Mélanie Suppan

**Affiliations:** 1 Media Engineering Institute, School of Management and Engineering Vaud, University of Applied Sciences and Arts of Western Switzerland, Avenue des Sports 20, Yverdon, 1400, Switzerland, 41 244592638; 2 Faculty of Biology and Medicine, University of Lausanne, Lausanne, Switzerland; 3 Division of Emergency Medicine, Department of Anesthesiology, Clinical Pharmacology, Intensive Care and Emergency Medicine, University of Geneva Hospitals and Faculty of Medicine, Geneva, Switzerland; 4 Division of Anesthesiology, Department of Anesthesiology, Clinical Pharmacology, Intensive Care and Emergency Medicine, University of Geneva Hospitals and Faculty of Medicine, Geneva, Switzerland

**Keywords:** physiological model, mathematical model, computer simulation, major incident management, emergency medicine, mass casualties, healthcare professional education, professional education, continuing education

## Abstract

**Background:**

Major incidents correspond to any situation where the location, number, severity, or type of casualties requires extraordinary resources. Major incident management must be efficient to save as many lives as possible. As any paramedic or emergency medical technician may unexpectedly have to respond to major incidents, regular training is mandatory. Those trainings usually include simulations. The vast majority of major incident simulations are limited by the fact that simulated patients do not evolve during the simulation, regardless of the time elapsed and treatment decisions. Therefore, most simulations fail to incorporate the critical temporal effect of decision-making.

**Objective:**

This study aimed to develop and validate a simplified mathematical model of physiology, capable of plausibly simulating the real-time evolution of several injuries.

**Methods:**

A modified version of the user-centered design framework, including a relevance, development, and validation phase, was used to define the development process of the physiological model. A 12-member design and development team was established, including prehospital physicians, paramedics, and computer scientists. To determine whether the developed model was clinically realistic, 15 experienced professionals working in the prehospital field participated in the validation phase. They were asked to rate clinical and physiological parameters according to a 5-point Likert scale ranging from 1 (impossible) to 5 (absolutely realistic).

**Results:**

The design and development team led to the development of the HUMAn model (Human is an Uncomplicated Model of Anatomy). During the relevance phase, the team defined the needed features of the model: clinically realistic, able to compute the evolution of prehospital vital signs, yet simple enough to allow real-time computation for several simulated patients on regular computers or tablets. During the development phase, iterations led to the development of a heart-lung-brain interaction model coupled to functional blocks representing the main anatomical body parts. During the validation phase, the evolution of nine simulated patients presenting pathologies devised to test the different systems and their interactions was assessed. Overall, clinical parameters of all patients had a median rating of 5 (absolutely realistic; IQR 4-5). Most (n=52, 96%) individual clinical parameters had a median rating of 5, the remainder (n=2, 4%) being rated 4. Overall physiological parameters of all patients had a median rating of 5 (absolutely realistic; IQR 3-5). The majority of individual physiological parameters (n=43, 79%) had a median rating of 5, with (n=9, 17%) rated 4, and only (n=2 ,4%) rated 3.

**Conclusions:**

A simplified model of trauma patient evolution was successfully created and deemed clinically realistic by experienced clinicians. This model should now be included in computer-based simulations and its impact on the teaching of major incident management assessed through randomized trials.

## Introduction

### Background and Importance

In the health sector, major incidents correspond to “any incidents where the location, number, severity or type of lives of casualties requires extraordinary resources” [1]. Major incidents are rare events that require efficient and effective management to save as many lives as possible [1,2]. The scarcity of these events and the impact they can have both on the victims and on society, as the impact such stressful situations may have on rescuers clinical performance [3] has led to the development of many different training modalities [4]. Thus, most prehospital providers are trained to manage major incidents through tabletop or computer-based simulations, practical exercises without casualties, or full-scale simulations [5].

These simulations all present limitations, some of which can often be addressed by using other forms of training. However, 1 important limitation is common to almost all major incident simulations and resides in the fact that simulated patients do not evolve over time [6]. Indeed, in most simulations, victims do not evolve at all regardless of the time elapsed and despite treatment and transport decisions. For instance, a simulated victim experiencing a massive internal hemorrhage (such as splenic rupture) will present identical vital signs throughout the simulation [7]. Consequently, most current simulations fail to incorporate the temporal effects of decision-making, which is a major characteristic of major incident management [8].

A simplified mathematical model of human physiology could be a solution for simulating victims’ evolution over time [9]. However, most currently available mathematical models of human physiology present limitations preventing their use in real-time major incident simulations. In 2005, Sacco et al [10] proposed a model using respiratory rate, pulse rate, and motor response (RPM) to predict victims’ survival probabilities. In this model, a single discrete modification of RPM parameters is made depending on whether the victims remains at the incident scene or is transported to a hospital. This change of RPM parameters is based on expert consensus. While insightful for predicting survival probabilities, this model does not enable to calculate the continuous time-based evolution of multiple physiological and clinical parameters, based on treatment decisions. Many others models are complex and ask for high processing power, making them incompatible with their use to simulate the synchronous evolution of several victims[11]. Furthermore, most models are not open source and cannot be embedded into third-party applications [12].

### Objectives

Our objective was to develop and validate a simplified physiological mathematical model capable of simulating the evolution of several casualties, in real time, while remaining realistic in the context of major events training.

## Methods

### General Design

A modified version of the user-centered design (UCD) framework proposed by Farao et al [13] was used to define the development process of the physiological model. The UCD framework is based on the 5 modes of design thinking (empathize, define, ideate, prototype, and test) [14], with the addition of the knowledge base mode of the information systems research framework [15].

**Figure 1.**
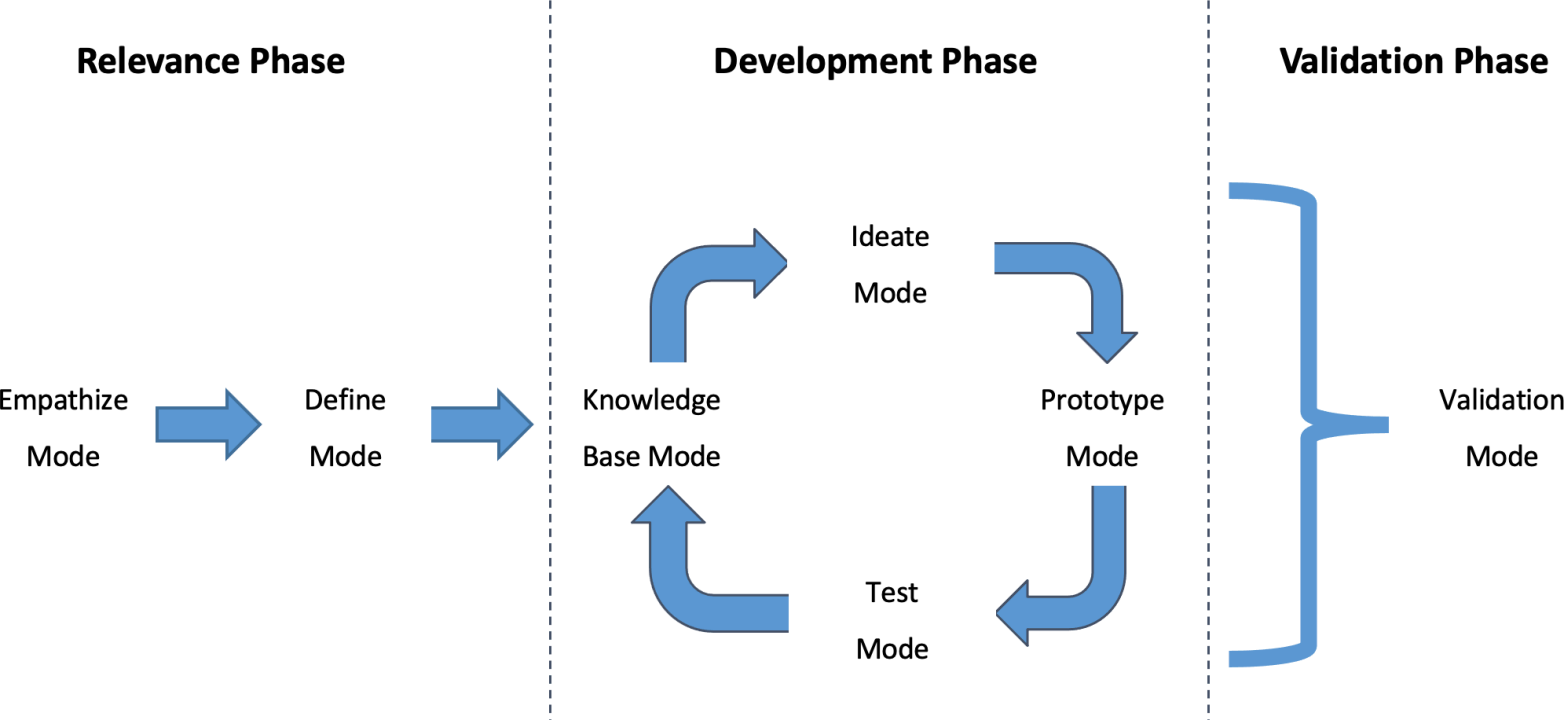
Framework used for the development and validation of the physiological model. The framework integrates the 6 modes of the user-centred design framework with the addition of a validation phase.

These 6 modes were sorted in 2 phases, the relevance phase and an iterative development phase. To validate the developed model, a validation phase was added. The overall resulting framework is summarized in Figure 1.

### Relevance Phase

The objective of the relevance phase was to identify the problem to be solved and define the overall goal for the development phase.

#### Empathize Mode

The goal of the empathize mode was to identify and understand the end-users and the context of use of the physiological model. Relevant elements of the co.LAB framework, which has been shown to have a positive impact on team collaboration when designing serious games [16,17], were used to determine the learning contexts where the physiological model could be used. In line with the philosophy of the co.LAB framework, end-users were included in the design process early on. A 12-member team was established, including 3 prehospital senior physicians and 3 advanced paramedics (all with extensive emergency medicine experience), 1 medical student, 1 game designer, 3 computer scientists, and 1 instructional designer. A total of 8 team members are coauthors of this article.

#### Define Mode

Based on results of the empathize mode, the define mode aimed at identifying the needed characteristics of the physiological model as well as basic technical requirements. This was achieved through several collaborative sessions between medical doctors and computer scientists.

### Development Phase

The objective of the development phase was to develop a conceptual physiological model, implement it into a software application, and test its ability to simulate the evolution of victims during a major incident.

#### Knowledge Base Mode

The knowledge base mode aimed at identifying existing solutions and reliable knowledge foundation for the development of the physiological model. First, Google Scholar and the regular Google search engine were used to identify already existing open-source computational models of physiology. Available models were assessed for their relevance against our objectives. Since no such model was found, a team of medical doctors and computer scientists collaborated to identify physiological and physiopathological formulas necessary to develop a model corresponding to the characteristics detailed through the define mode. Searches were done in research databases, textbooks and grey literature. After the first iteration, further searches were based on the results of the test mode and relevant formulas inserted accordingly.

#### Ideate Mode

The ideate mode was used to define the combination of formulas needed to develop a simple yet clinically plausible physiological model. It also included ideation for technological solutions for the development of the software simulation. Furthermore, this mode was used to investigate and define the algorithmic implementation of formulas in the software. This was carried out through brainswarming sessions involving medical doctors, computer scientists and paramedics [18].

#### Prototype Mode

Basic implementations of formulas were first done on spreadsheets and then implemented into a full software application. Computational models of independent physiological systems (respiratory, cardiovascular, and brain) were first developed. Once deemed realistic enough by the development team, interactions between these systems were implemented. After ensuring that interactions were appropriate under normal physiological conditions, elements required to simulate injuries were progressively added and tested.

#### Test Mode

Each prototype was tested by a panel of doctors and paramedics, all belonging to the development team. Tests were first conducted to assess the plausibility of the simulated independent physiological systems and, then, the simultaneous behavior of those systems. Situations involving single, then multiple injuries were then tested. Tests consisted of an evaluation of the plausibility of the evolution of clinical and physiological parameters.

### Validation Phase

During the validation phase, relevant elements of Eysenbach’s Checklist for Reporting Results of Internet E-Surveys (CHERRIES) [19] have been used to report the results.

#### Design and Study Sequence

To determine whether the developed model was clinically realistic, 15 experienced prehospital professionals (5 advanced paramedics and 10 senior registrars working in ground and helicopter emergency medical services, none of them being part of the development team) were invited to rate clinical and physiological parameters according to a 5-point Likert scale ranging from 1 (impossible) to 5 (absolutely realistic).

After a short briefing regarding the study’s aims, these experts were asked to create a personal account on an web testing platform. The top of the page displayed the patient’s characteristics (sex, age, height, and weight), their pathology, and their time of death if the model predicted it would happen within 4 hours if left untreated. All experts were clearly informed that they would have to conceptualize how patient parameters would evolve if they did not receive any medical treatment.

Clinical (oxygen saturation, respiratory rate, heart rate, mean arterial pressure, Glasgow coma scale, and ability to walk) and physiological (alveolar volume, PaO2, PaCO2, blood volume, stroke volume, and intracranial pressure) parameters were displayed at 5 different time points. The first time point (T0) corresponds to the initial state, just before the simulated patient was ascribed a specific pathology. The last point (T4) corresponds to the time of death of the simulated patient, with a maximum T4 equals to 4 hours if the survival time was more than 4 hours. To assess the plausibility of the evolution of the parameters, intermediate values between T0 and T4 were presented at times corresponding to 25% (T1), 50% (T2), and 75% (T3) of T4.

Although the use of relative times for T1 to T4 leads to an assessment of clinical and physiological parameters at different absolute times for each patient, we chose this approach because it enable us to assess the plausibility of the evolution of the parameters at similar relative times to death. In order to give the complete information to the experts who assessed the model, the absolute values of T1, T2, T3, and T4 were displayed in the form of “hours : minutes : seconds” for each simulated patient.

**Figure 2.**
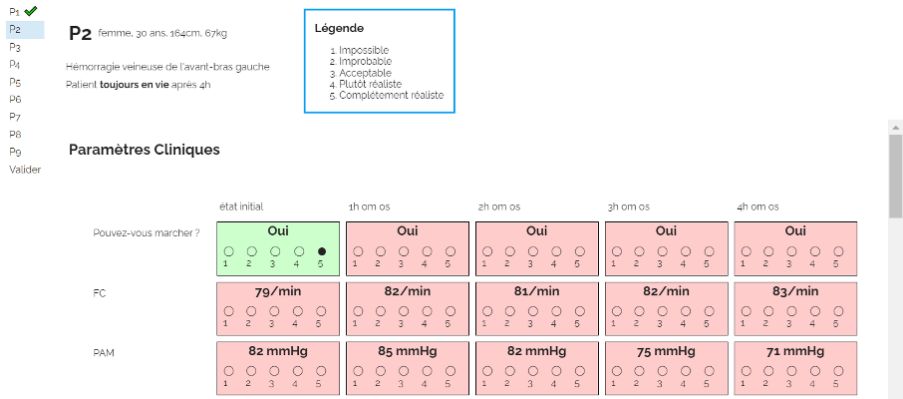
Screenshot of the web assessment form. A summary of the simulated patients to be evaluated is displayed on the left side. The patient’s characteristics and the Likert scale legend are shown on the top of the page. Each parameter is shown on a single row, and each column represents a specific timepoint. Once the expert has chosen a rating, the background turns to green.

Figure 2 shows a screenshot of the system used to display parameters and enable participants to rate them. In this particular case, the patient was expected to survive more than 4 hours. Thus the last time point is equal to 4 hours and intermediate time points correspond to 1, 2, and 3 hours.

#### Statistical Analysis

Data were extracted to a .csv file and imported in Stata (version 17.0. StataCorp) for curation and statistical analysis. All clinical and physiological parameters were individually tested at each time point to determine their perceived reliability. Their overall perceived reliability was then assessed. Clinical parameters (oxygen saturation, respiratory rate, heart rate, mean arterial pressure, Glasgow coma scale, and ability to walk) were grouped and tested together at each timepoint and overall. The same procedure was applied to physiological parameters (alveolar volume, PaO_2_, PaCO_2_, blood volume, stroke volume, and intracranial pressure). Results are reported as median (IQR) to better reflect the definitions of the Likert scale.

All clinical and physiological parameters were individually tested at each time point to determine their perceived reliability. Their overall perceived reliability was then assessed. Clinical parameters (SpO_2_, respiratory rate, heart rate, mean arterial pressure, Glasgow coma scale, ability to walk) were grouped and tested together at each timepoint and overall. The same procedure was applied to physiological parameters (alveolar volume, PaO_2_, PaCO_2_, blood volume, stroke volume, intracranial pressure).

### Ethical Considerations

Since the participants who took part in the validation phase were all professionals working in the prehospital field, their participation did not fall within the scope of the Swiss Federal Act on Research involving Human Beings (HRA, RS 810.30). All the participants agreed to take part in this study without any form of compensation, and no incentive other than pure scientific interest was used to promote participation. The results of the evaluation phase were anonymized to ensure the confidentiality of participants’ assessments.

All the participants agreed to take part in this study without any form of compensation, and no incentive other than pure scientific interest was used to promote participation. The results of the evaluation phase were anonymized to ensure the confidentiality of participants’ assessments.

## Results

### Relevance Phase

#### Empathize Mode

Several collaborative design sessions were organized. All collaborative sessions were held in the presence of at least 3 members of the overall team, with the medical and game development professions systematically represented. At the end of each session, ideas were discussed and synthesized.

#### Define Mode

The team agreed on the characteristics of the physiological model that was to be developed. The most important feature was that the model was to be clinically realistic. This entailed that it should include all the elements required to compute vital signs and their evolution according to the presence of pathologies usually encountered in major incidents, such as hemorrhages, burns, pneumothoraxes, and head traumas. In addition, the model was to be simple enough to allow real-time computation of all relevant vital parameters for several simulated patients simultaneously. It was also decided that the system should be able to randomly create simulated patients. Taking all these elements into account, it was determined that the model would be limited to the characteristics outlined in Table 1.

**Table 1.** Characteristics of simulated patients.

Characteristics			Values
Age (years), range			16-100
Sex (boolean)			Male or female
BMI (kg/m^2^), mean (SD)			23 (3)
Height (cm), mean (SD)			
Man			178.4 (7.6)
Woman			164.7 (7.1)

### Development Phase

#### Knowledge Base Analysis

When models were readily available, knowledge base analysis was a straightforward process and all relevant references were rapidly included. Several examples are given in Table 2.

**Table 2.** Examples of data and formulae obtained through knowledge base analysis.

Variable [study]		Data and formulae
Ideal weight (kg) [20]		
Man		50+0.9 (kg/cm)*(height [cm]-152.4 [cm])
Woman		45.5+0.9 (kg/cm)*(height [cm]-152.4 [cm])
Blood volume (mL) [21]		70*ideal weight (kg)
Dead space (mL) [22]		2.2 (mL/kg)*ideal weight (kg)
Respiratory quotient [23]		0.84
Cerebral blood flow (L/min) [24]		0.5 (L*min/kg)*1.4 (kg)
Cerebral perfusion pressure (mmHg) [25]		Mean arterial pressure – intracranial pressure

#### Ideate Mode

Since data regarding patient evolution according to specific injuries were often lacking, the mathematical formulae controlling the evolution of physiological parameters in the presence of such injuries had to be developed through a collaboration between clinicians and computer scientists. These formulae were progressively refined through multiple iterations. Table 3 shows the steps used to compute systolic and diastolic blood pressures according to volemia, sympathetic nervous system activation, pericardic and pleural pressures, and cardiac contractility. While many parameters are taken into account, several were either simplified or used as constants. Nevertheless, the model enables the inclusion of future updates to simulate currently simplified elements such as vascular resistance.

**Table 3.** Steps used to determine diastolic and systolic blood pressure.

Step and parameters		Data and formulae	Comment
Initial data			
Blood volume (mL)		70*weight [21]	—^a^
Systemic vascular resistance (mmHg*min/L)		13 [26]	—
Initial EDV^b^_Initial_ (mL)		120	—
Effect of blood volume on EDV (ΔEDV_Volemia_) (mL)		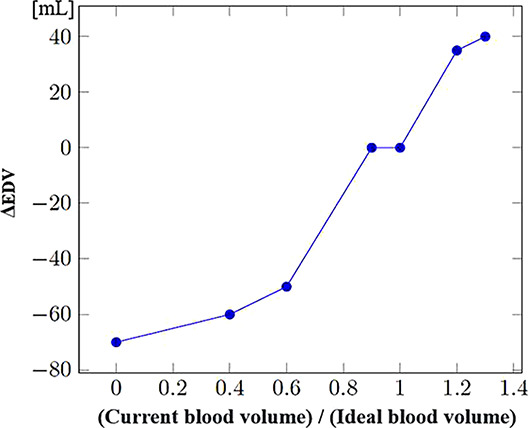	EDV is reduced in the event of blood loss (hypovolemia), increased in the event of hypervolemia. The model allows it to vary between +40ml and −70ml according to this parameter. (model-specific approximations rather than established formulas)
Effect of pericardial pressure on EDV (ΔEDV_PPericardial_) (mL)		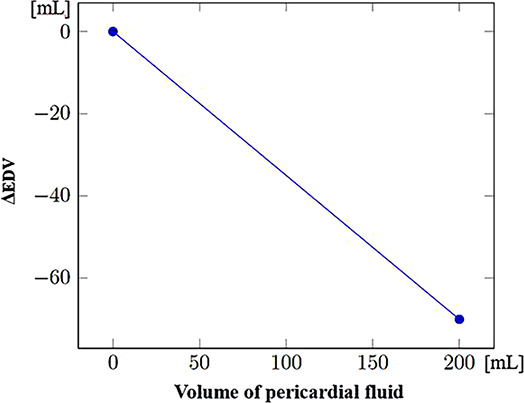	A pericardial effusion causes fluid to accumulate in the pericardium. Consequently, EDV decreases along with the increase in pericardial fluid. (model-specific approximations rather than established formulas)
Effect of pleural pressure on EDV (ΔEDV_PPleural_) (mL)		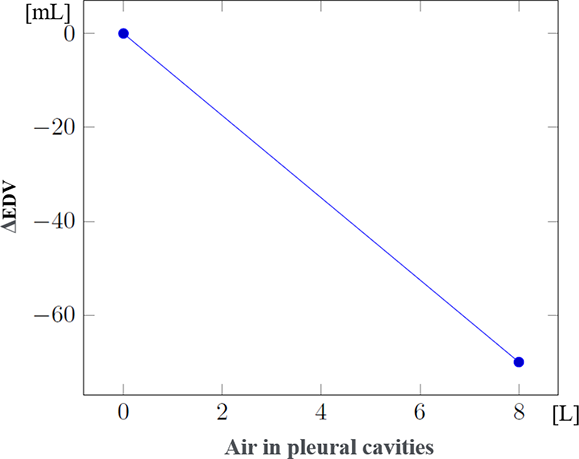	This simulates a limitation in heart filling consecutive to an increase in pleural cavity pressure. (model-specific approximations rather than established formulas)
Effect of sympathetic nervous system activation on EDV (ΔEDV_Sympathetic_) (mL)		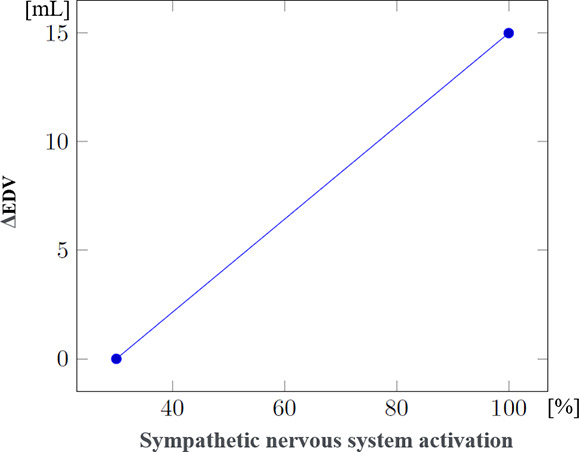	This is a very simplified approximation designed to simulate the increase in venous return consecutive to sympathetic activation.(model-specific approximations rather than established formulas)
Final EDV (EDV) (mL)		EDV =EDV_Initial_ + ΔEDV_Volemia_ + ΔEDV_PPericardial_ + ΔEDV_PPleural +_ ΔEDV_Sympathetic_	—
ESV^c^ (mL)		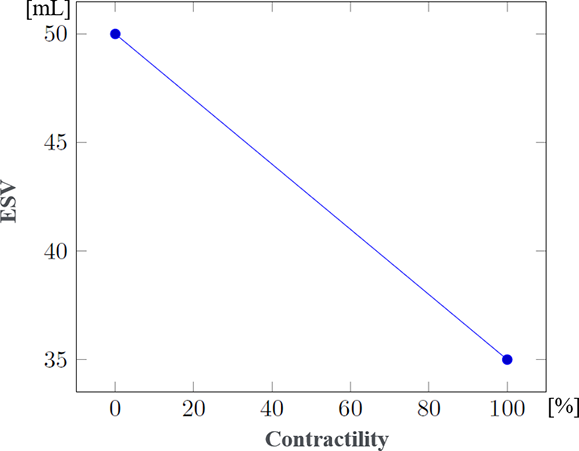	(model-specific approximations rather than established formulas)
SV^d^ (ml)		SV = EDV – ESV [27]	—
CO^e^ (L/min)		CO = SV * HR * 1000 [27]	—
Left ventricular pressure (mmHg)		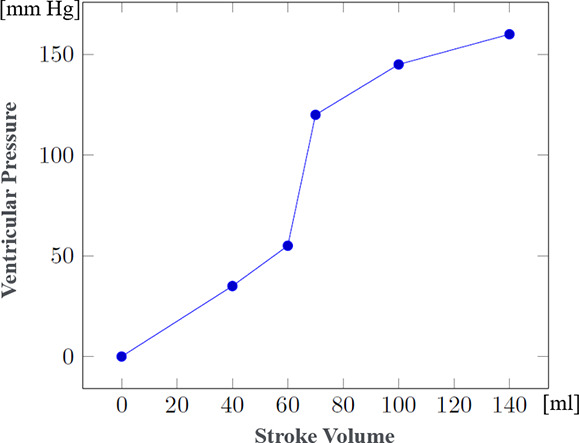	(model-specific approximations rather than established formulas)
MAP^f^_th_ (mmHg)		MAP_th_ = CO * SVR [27]	—
MAP^g^_eff_ (mmHg)		If MAP_th_< VP: MAPeff = MAP_th_ If MAP_th_ ≥ VP: MAPeff = VP	MAP cannot be higher than VP (model-specific approximations rather than established formulas)
Actual CO (CO _eff_) (L/min)		If MAP_th_< VP: CO _eff_ = MAP_eff_ / SVR Else: CO _eff_ = CO	Adjustment of CO if theoretical MAP cannot be achieved (model-specific approximations rather than established formulas)
Calculation of DBP^h^ and SBP^i^ (mmHg)		DBP = 6/7 * MAP_eff_ SBP = 3/2 * MAP_eff_	(model-specific approximations rather than established formulas)

a —: not applicable.

b EDV: end-diastolic volume.

c ESV: end-systolic volume.

d SV: stroke volume.

e CO: cardiac output.

f MAP_th_: theoretical mean arterial pressure.

g MAP_eff_: actual MAP.

h DBP: diastolic blood pressure.

i SBP: systolic blood pressure.

Brainswarming processes led to retain two other main concepts for the development of the application: the heart-lung-brain interaction model and the concept of functional blocks. The heart-lung-brain interaction model was decided since these 3 organs are responsible for almost all the vital signs linked to the ABC (airways, breathing, and circulation) evaluation performed by prehospital providers. Although other organs, such as the liver and the kidney, are of course vital, their function does not significantly alter vital signs in the immediate aftermath of a major incident.

**Figure 3.**
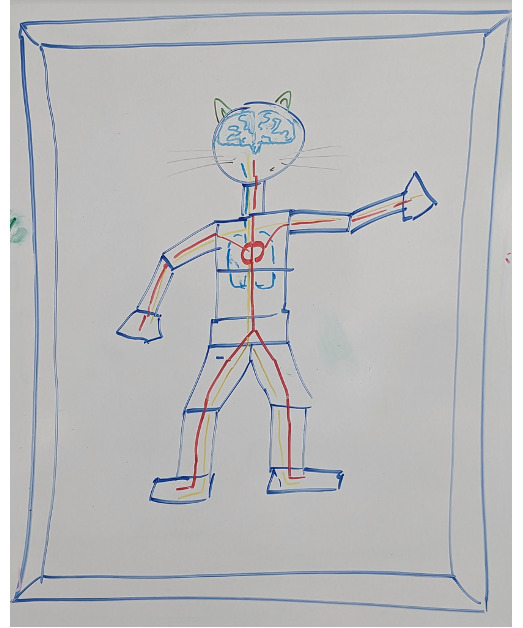
Original drawing illustrating the concept of functional blocks. These blocks are used to divide the body into anatomical subsections, allowing traumas to be created at different locations thus enabling localized treatments.

The concept of functional blocks (Figure 3) was elaborated to provide more information regarding the neurological status of simulated patients and to create hemorrhages at different levels, with different blood flows, thereby allowing specific treatment options.

#### Prototype Mode

Prototypes were developed and tested on Wegas, an open-source web-based serious game authoring and execution platform [28]. Wegas source code can be downloaded on Github [29]. The model source code is freely available under a Creative Commons 4.0 CC-BY-NC licence. The source code can be downloaded on Github [30]. The model’s source code is in English. At the implementation level, user interfaces are designed to be multilingual. The current version of the implementation is in French, but other languages may be added in the future.

The model source code is freely available under a Creative Commons 4.0 CC-BY-NC licence. The source code can be downloaded on Github [30]. The model’s source code is in English. At the implementation level, user interfaces are designed to be multilingual. The current version of the implementation is in French, but other languages may be added in the future.

#### Test Mode

All prototypes were tested by paramedics and physicians belonging to the development team. Their comments were used to progressively refine the model through new iterations of the knowledge base analysis and ideate modes.

### Validation Phase

In total, 15 experienced prehospital professionals (5 advanced paramedics and 10 senior registrars working in ground and helicopter emergency medical services) were invited to participate in the validation study. All of them agreed to participate. Their characteristics are detailed in Table 4.

**Table 4.** Characteristics of the prehospital experts.

Characteristics		Overall (N=15)	Physicians (n=10)	Paramedics (n=5)
Gender, n (%)				
Man		11 (73)	6 (60)	5 (100)
Woman		4 (27)	4 (40)	0 (0%)
Other		0 (0)	0 (0)	0 (0%)
Age (years), mean (SD)		40 (6)	39 (5)	41 (7)
Postgraduate clinical experience (years), mean (SD)		11 (7)	10 (6)	13 (9)
Prehospital experience (years), mean (SD)		10 (8)	7 (7)	16 (5)

In order to test the different systems and their interactions, 9 simulated patients were automatically generated, with different personal characteristics. We then ascribed a specific pathology to each patient. No patient was ascribed more than 1 pathology. The characteristics of patients and ascribed pathologies are detailed in Table 5.

**Table 5.** Characteristics of the patients automatically generated by the simulator.

Patient	Sex (M/F)	Age (years)	Height (cm)	Weight (kg)	Ascribed pathology	Time of death (seconds)
Patient 1	M^a^	45	159	64	Massive internal hemorrhage (eg, ruptured spleen)	2700
Patient 2	F^b^	30	164	67	Veinous hemorrhage of the left forearm	>14,400
Patient 3	M	42	182	86	Traumatic cardiac tamponade	8850
Patient 4	F	63	161	53	Complete left pneumothorax	>14,400
Patient 5	F	46	168	68	Circular second-degree burns of the face and neck	5735
Patient 6	F	50	170	61	Complete circumferential third-degree burn of the chest	5670
Patient 7	F	32	161	62	Head trauma with cerebral hemorrhage	4745
Patient 8	F	61	164	69	Atlanto-occipital dislocation	345
Patient 9	M	44	168	57	Unstable, displaced cervical fracture between C5 and C7	>14,400

a M: male.

b F: female.

The validation team assessed each patient clinical and physiological parameters from T0 to T4. The initial and final states of all patients had a median rating of 5 (absolutely realistic) for both clinical (Table 6) and physiological (Table 7) parameters. The overall median rating was also 5 for each individual patient. Detailed assessments, by patient and time period, are displayed in Tables6  7. Ratings as mean (SD) are available in Multimedia Appendix 1.

**Table 6.** Assessment of the simulated patients’ overall clinical parameters.

Patient	T0, median (IQR)	T1, median (IQR)	T2, median (IQR)	T3, median (IQR)	T4, median (IQR)	Overall, median (IQR)
Patient 1	5 (5-5)	5 (4-5)	4 (4-5)	5 (4-5)	5 (5-5)	5 (4-5)
Patient 2	5 (5-5)	5 (5-5)	5 (5-5)	5 (4-5)	5 (4-5)	5 (5-5)
Patient 3	5 (5-5)	5 (4-5)	4 (3-5)	5 (4-5)	5 (5-5)	5 (4-5)
Patient 4	5 (5-5)	5 (4-5)	5 (4-5)	5 (4-5)	5 (4-5)	5 (4-5)
Patient 5	5 (5-5)	5 (4-5)	5 (3-5)	4 (3-5)	5 (5-5)	5 (4-5)
Patient 6	5 (5-5)	5 (4-5)	5 (3-5)	4 (3-5)	5 (5-5)	5 (4-5)
Patient 7	5 (5-5)	5 (4-5)	4 (3-5)	5 (4-5)	5 (5-5)	5 (4-5)
Patient 8	5 (5-5)	5 (3-5)	5 (3-5)	5 (3-5)	5 (5-5)	5 (4-5)
Patient 9	5 (5-5)	5 (4-5)	5 (4-5)	5 (4-5)	5 (4-5)	5 (4-5)

**Table 7.** Assessment of the simulated patients’ overall physiological parameters.

Patient	T0, median (IQR)	T1, median (IQR)	T2, median (IQR)	T3, median (IQR)	T4, median (IQR)	Overall, median (IQR)
Patient 1	5 (4-5)	5 (4-5)	4 (3-5)	4 (3-5)	5 (4-5)	5 (3-5)
Patient 2	5 (5-5)	5 (5-5)	5 (5-5)	5 (4-5)	5 (4-5)	5 (5-5)
Patient 3	5 (5-5)	5 (4-5)	4 (3-5)	4 (3-5)	5 (5-5)	5 (4-5)
Patient 4	5 (5-5)	5 (4-5)	5 (4-5)	5 (4-5)	5 (4-5)	5 (4-5)
Patient 5	5 (5-5)	5 (4-5)	5 (4-5)	5 (4-5)	5 (5-5)	5 (4-5)
Patient 6	5 (5-5)	5 (4-5)	5 (4-5)	5 (3-5)	5 (5-5)	5 (4-5)
Patient 7	5 (5-5)	5 (5-5)	5 (4-5)	5 (4-5)	5 (5-5)	5 (5-5)
Patient 8	5 (5-5)	5 (4-5)	5 (4-5)	5 (4-5)	5 (5-5)	5 (4-5)
Patient 9	5 (5-5)	5 (3-5)	5 (3-5)	5 (3-5)	5 (3-5)	5 (3-5)

Overall clinical parameters include oxygen saturation, respiratory rate, heart rate, mean arterial pressure, Glasgow coma scale, and ability to walk. T4 corresponds to the time of death of the simulated patient (T4 equals 4 hours if the survival time was more than 4 hours). T1 to T4 correspond respectively to 25%, 50%, and 75% of T4. Results are presented as “median (IQR).”

Overall physiological parameters include alveolar volume, PaO2, PaCO2, blood volume, stroke volume, and intracranial pressure. T4 corresponds to the time of death of the simulated patient (T4 equals 4 hours if the survival time was more than 4 hours). T1 to T4 correspond respectively to 25%, 50%, and 75% of T4. Results are presented as “median (IQR).”

The overall median rating for each specific clinical (Table 8) and physiological (Table 9) parameter was 5 (absolutely realistic). Further results are available in Multimedia Appendix 1.

**Table 8.** Assessment of the simulated patients’ clinical parameters.

Patient	SpO_2,^a^_ median (IQR)	RR,^b^ median (IQR)	HR,^c^ median (IQR)	MAP,^d^ median (IQR)	GCS,^e^ median (IQR)	Walks,^f^ median (IQR)
Patient 1	5 (4-5)	5 (4-5)	5 (3-5)	5 (4-5)	5 (4-5)	5 (4-5)
Patient 2	5 (5-5)	5 (4-5)	5 (4-5)	5 (5-5)	5 (5-5)	5 (5-5)
Patient 3	5 (5-5)	5 (5-5)	5 (4-5)	5 (4-5)	5 (4-5)	5 (4-5)
Patient 4	4 (4-5)	5 (4-5)	5 (4-5)	5 (4-5)	5 (5-5)	5 (4-5)
Patient 5	5 (4-5)	5 (4-5)	5 (3-5)	5 (4-5)	5 (5-5)	5 (4-5)
Patient 6	5 (4-5)	5 (4-5)	5 (4-5)	5 (4-5)	5 (5-5)	5 (3-5)
Patient 7	5 (5-5)	5 (4-5)	5 (4-5)	5 (4-5)	5 (4-5)	5 (4-5)
Patient 8	5 (4-5)	5 (5-5)	5 (4-5)	5 (4-5)	4 (2-5)	5 (5-5)
Patient 9	5 (4-5)	5 (4-5)	5 (4-5)	5 (4-5)	5 (5-5)	5 (5-5)
Overall	5 (4-5)	5 (4-5)	5 (4-5)	5 (4-5)	5 (4-5)	5 (4-5)

a SpO_2_: oxygen saturation.

b RR: respiratory rate.

c HR: heart rate.

d MAP: mean arterial pressure.

e GCS: Glasgow Coma Scale.

f Ability to walk.

**Table 9.** Assessment of the simulated patients’ physiological parameters. Results are presented as “median (IQR)”.

	PaO_2,^a^_ median (IQR)	PaCO_2,^b^_ median (IQR)	Alveolar volume, median (IQR)	Blood volume, median (IQR)	Stroke volume, median (IQR)	ICP,^c^ median (IQR)
Patient 1	4 (3-5)	4 (2-5)	4 (3-5)	4 (3-5)	5 (4-5)	5 (5-5)
Patient 2	5 (5-5)	5 (4-5)	5 (5-5)	5 (4-5)	5 (4-5)	5 (5-5)
Patient 3	5 (4-5)	4 (3-5)	5 (4-5)	5 (4-5)	5 (4-5)	5 (5-5)
Patient 4	4 (4-5)	4 (4-5)	4 (4-5)	5 (5-5)	5 (4-5)	5 (5-5)
Patient 5	5 (4-5)	5 (4-5)	5 (4-5)	5 (5-5)	5 (5-5)	5 (5-5)
Patient 6	5 (4-5)	5 (4-5)	5 (4-5)	5 (4-5)	5 (5-5)	5 (5-5)
Patient 7	5 (5-5)	5 (4-5)	5 (5-5)	5 (5-5)	5 (5-5)	5 (4-5)
Patient 8	5 (4-5)	5 (4-5)	5 (5-5)	5 (5-5)	5 (3-5)	5 (5-5)
Patient 9	4 (3-5)	3 (2-5)	3 (3-5)	5 (5-5)	5 (4-5)	5 (5-5)
Overall	5 (4-5)	5 (3-5)	5 (4-5)	5 (4-5)	5 (4-5)	5 (5-5)

a PaO_2_: arterial partial pressure of O_2_.

b PaCO_2_: arterial partial pressure of CO_2_.

c ICP: intracranial pressure.

## Discussion

### Principal Findings

A simplified model of trauma patient evolution was successfully created using the modified version of the user-centered design framework and was deemed clinically realistic by experienced prehospital physicians and paramedics. The diversity of simulated clinical cases, which included nervous, cardiovascular and respiratory pathologies, and its suitability for real-time calculations makes this model a good candidate for inclusion in major incident simulations. Many kinds of simulations could be considered, computer-based table-tops and serious games, practical exercises without casualties, and even full-scale simulations. In this latter case, tablets could be used to display the vital parameters of simulated patients.

All clinical and physiological parameters had an overall median rating of 5 (absolutely realistic). Nevertheless, some parameters had lower median ratings when specific patients were analyzed. For instance, the oxygen saturation values of patient 4, who presented a complete left pneumothorax, had a median rating of 4 (rather realistic). Several hypotheses could explain these findings. First, even though an iterative process was followed to progressively refine the evolution of specific parameters, the current model is still an approximation of actual human physiology. Thus, further iterations may enable further improvements. Second, prehospital clinicians quickly manage airway and breathing issues when responding to critical situations. Therefore, these professionals have never witnessed the evolution of patients left untreated and could find it harder to conceive how vital and physiological parameters would develop in such cases. Finally, given the inherent variability in injury presentations and the subjective nature of textual descriptions, there is potential for variation in interpretation among prehospital providers. This inter-rater variability may contribute to the differing assessments of injury realism.

Other models have previously been developed [31], none of which were however deemed suitable for our purpose, that is, real-time simulation of multiple trauma patients with realistic heart-lung-brain interactions. While models such as HumMod and Biogears provide realistic, scientifically grounded models of patient evolution [32,33], their complexity usually requires considerable computational power, making them hardly suitable for major incident simulations. In addition, and although many physiological processes are supported by clear scientific literature and rules, the lack of data regarding most prehospital injuries had to be compensated for by clinical experience. The inclusion of senior prehospital physicians and paramedics since inception has thus enabled the creation of a model realistic enough to simulate the evolution of trauma patients during a major incident.

The model developed through this study presents the advantage of being based on an anatomically realistic model made of several blocks simulating the different body compartments which could be involved in case of injury. Neural and vascular lesions are thus easy to simulate, and these blocks could be divided up further if needed. Furthermore, other organ systems could be added according to the end-user’s requirements: for instance, adding clearance mechanisms could prove useful to the management of major incidents involving hazardous materials. Conversely, disabling or removing systems uninvolved in the simulated situation could save processing power.

Simulating the outcome of patients involved in major incidents could prove useful in several ways. Including an evolutive model in computer-based simulations could help ambulance and medical commanders better understand the impact of certain decisions, such as the use of human resources for treatment rather than for triage. The importance of accurate triage cannot be overstated and methods based on patient evolution should be promoted [9]. In this regard, this physiological model could be used to compare different triage algorithms among different populations. Indeed, some professional categories may be more efficient than others depending on the tool provided [34].

### Limitations

This model is not without limitations. The most important and most obvious limitation is the lack of pediatric-specific formulae, which prevents the generation of patients aged 16 years or younger. Another significant limitation is the absence of treatment-specific effects, which would be necessary to assess the effect of human and material resources through computer-based simulations. Since physiological and clinical parameters of simulated patients were assessed at relative intermediate time points (25%, 50%, and 75%) of death time, the absolute times (hours: minutes: seconds) of those intermediate points varied across simulated patients. This may complicate assessing patients’ conditions over time. However, to enhance clarity, the absolute value of each intermediate time point was displayed on the interface. In addition, experts were informed about the calculation method for intermediate time points before starting the evaluation of parameters. While the overall scores, both for clinical and physiological parameters, show the high realism of the validation study, overall scores could potentially hide specific inconsistencies. However, Q1 and Q3 values are mostly close to median values. The plausibility of simulated patients’ parameters has been assessed by only 15 experts. Given the overall score used in the assessment, the relatively small number of experts who evaluated the model may not have highlighted individual inconsistencies. Finally, the current version of this simplified physiological model was only developed to simulate traumas. Thus, other causes of major incidents, such as those linked to hazardous materials, cannot be simulated without further developments.

Another significant limitation is the absence of treatment-specific effects, which would be necessary to assess the effect of human and material resources through computer-based simulations.

Since physiological and clinical parameters of simulated patients were assessed at relative intermediate time points (25%, 50%, 75%) of death time, the absolute times (hours: minutes: seconds) of those intermediate points varied across simulated patients. This may complicate assessing patients’ conditions over time. However, to enhance clarity, the absolute value of each intermediate time point was displayed on the interface. Additionally, experts were informed about the calculation method for intermediate time points before starting the evaluation of parameters.

While the overall scores, both for clinical and physiological parameters, show the high realism of the validation study, overall scores could potentially hide specific inconsistencies. However, Q1 and Q3 values are mostly close to median values.

The plausibility of simulated patients’ parameters has been assessed by only 15 experts. Given the overall score used in the assessment, the relatively small number of experts who evaluated the model may not have highlighted individual inconsistencies.

Finally, the current version of this simplified physiological model was only developed to simulate traumas. Thus, other causes of major incidents, such as those linked to hazardous materials, cannot be simulated without further developments.

### Perspectives

Embedding this model in computer-based simulations and serious games designed to teach major incident management should now be considered. The inclusion of evolutive pathologies could help determine the most efficient ways of dealing with specific major incident situations and prepare health professionals to deal with the stress of managing such events [3]. Further developments should also be contemplated. These should help address several limitations, including the unavailability of pediatric patients and the inclusion of treatment specific effects.

### Conclusion

A simplified model of trauma patient evolution was successfully created and deemed clinically realistic by experienced clinicians. This model should now be included in computer-based simulations and its impact on the teaching of major incident management assessed through randomized trials.

## Supplementary material

10.2196/66201 Multimedia Appendix 1 Overall results of the validation phase, with ratings as mean (SD).

## Abbreviations

CHERRIES: Checklist for Reporting Results of Internet E-Surveys
RPM: respiratory rate, pulse rate, and motor response
UCD: user-centered design
