# Supplementary material for: HUMAn, a Real-Time Evolutive Patient Model for Major Incident Simulation: Development and Validation Study
Source: JMIR Form Res. 2025 Mar 7;9:e66201. doi: 10.2196/66201 (PMC11908467; doi:10.2196/66201)
Supplement: Multimedia Appendix 1 [file formative-v9-e66201-s001.docx]

Multimedia Appendix #2 – Supplementary Tables

|  | T0 | T1 | T2 | T3 | T4 | Overall |
| --- | --- | --- | --- | --- | --- | --- |
| Patient 1 | 4.7±0.9 | 4.2±1.0 | 4.2±0.8 | 4.3±0.9 | 5.0±0.0 | 4.5±0.9 |
| Patient 2 | 4.9±0.4 | 4.8±0.5 | 4.6±0.9 | 4.4±1.1 | 4.2±1.1 | 4.6±0.9 |
| Patient 3 | 4.9±0.4 | 4.4±1.1 | 3.9±1.3 | 4.2±1.1 | 5.0±0.1 | 4.5±1.0 |
| Patient 4 | 4.7±0.9 | 4.4±0.9 | 4.3±1.1 | 4.2±1.2 | 4.1±1.3 | 4.3±1.1 |
| Patient 5 | 4.7±0.8 | 4.4±0.9 | 4.2±1.1 | 4.0±1.1 | 5.0±0.0 | 4.5±0.9 |
| Patient 6 | 4.8±0.6 | 4.3±1.0 | 4.1±1.2 | 4.0±1.2 | 5.0±0.1 | 4.4±1.0 |
| Patient 7 | 4.9±0.4 | 4.3±1.0 | 3.9±1.1 | 4.4±1.1 | 5.0±0.1 | 4.5±0.9 |
| Patient 8 | 4.5±1.1 | 4.0±1.3 | 3.9±1.4 | 3.9±1.3 | 5.0±0.0 | 4.3±1.2 |
| Patient 9 | 4.7±0.8 | 4.4±0.9 | 4.4±0.9 | 4.4±0.9 | 4.4±0.9 | 4.5±0.9 |

**Supplementary Table 1.** Assessment of the simulated patients’ **overall clinical parameters** (SpO_2_, respiratory rate, heart rate, mean arterial pressure, Glasgow coma scale, ability to walk) with means±SD.

|  | T0 | T1 | T2 | T3 | T4 | Overall |
| --- | --- | --- | --- | --- | --- | --- |
| Patient 1 | 4.5±0.9 | 4.2±1.0 | 3.7±1.2 | 3.7±1.1 | 4.2±1.2 | 4.1±1.1 |
| Patient 2 | 4.8±0.6 | 4.6±0.8 | 4.6±0.9 | 4.5±0.9 | 4.4±1.1 | 4.6±0.9 |
| Patient 3 | 4.8±0.6 | 4.4±1.1 | 3.9±1.3 | 3.9±1.3 | 4.6±1.0 | 4.3±1.1 |
| Patient 4 | 4.7±0.6 | 4.4±0.9 | 4.3±1.0 | 4.3±1.0 | 4.3±1.1 | 4.4±0.9 |
| Patient 5 | 4.8±0.5 | 4.7±0.7 | 4.5±0.8 | 4.4±0.9 | 4.7±0.7 | 4.6±0.8 |
| Patient 6 | 4.8±0.6 | 4.6±0.7 | 4.4±0.9 | 4.2±1.1 | 4.8±0.6 | 4.5±0.8 |
| Patient 7 | 4.8±0.5 | 4.6±0.7 | 4.5±0.8 | 4.3±1.0 | 4.7±0.8 | 4.6±0.8 |
| Patient 8 | 4.6±0.8 | 4.4±1.0 | 4.3±1.0 | 4.4±1.0 | 4.7±0.8 | 4.5±0.9 |
| Patient 9 | 4.8±0.6 | 4.0±1.3 | 4.0±1.3 | 4.0±1.2 | 4.0±1.2 | 4.2±1.2 |

**Supplementary Table 2.** Assessment of the simulated patients’ **overall physiological parameters** (alveolar volume, PaO2, PaCO2, blood volume, stroke volume, intracranial pressure) with means±SD.

|  | T0 | T1 | T2 | T3 | T4 | Overall |
| --- | --- | --- | --- | --- | --- | --- |
| Patient 1 | 4.4±1.2 | 3.7±1.3 | 3.9±1.0 | 4.0±0.9 | 5.0±0.0 | 4.2±1.1 |
| Patient 2 | 4.8±0.6 | 4.6±0.8 | 4.1±1.2 | 4.3±1.0 | 4±1.1 | 4.4±1.0 |
| Patient 3 | 4.9±0.3 | 4.2±1.3 | 4.0±1.1 | 4.1±1.0 | 5.0±0.0 | 4.5±0.9 |
| Patient 4 | 4.7±0.8 | 4.5±0.9 | 4.1±1.2 | 3.9±1.3 | 3.9±1.4 | 4.2±1.2 |
| Patient 5 | 4.4±1.3 | 3.7±1.2 | 3.5±1.3 | 4.0±1.1 | 5.0±0.0 | 4.1±1.2 |
| Patient 6 | 4.7±0.8 | 3.7±1.3 | 3.5±1.3 | 4.1±1.1 | 5.0±0.0 | 4.2±1.2 |
| Patient 7 | 4.9±0.4 | 4.3±1.0 | 4.3±1.0 | 4.6±0.9 | 5.0±0.0 | 4.6±0.8 |
| Patient 8 | 4.7±0.9 | 4.3±0.8 | 4.1±1.0 | 3.9±1.2 | 5.0±0.0 | 4.4±0.9 |
| Patient 9 | 4.7±0.7 | 4.2±0.9 | 4.3±1.0 | 4.3±1.0 | 4.2±0.9 | 4.3±0.9 |

**Supplementary Table 3.** Assessment of the simulated patients’ **heart rate** at each time period.

|  | T0 | T1 | T2 | T3 | T4 | Overall |
| --- | --- | --- | --- | --- | --- | --- |
| Patient 1 | 4.5±1.2 | 4.2±1.0 | 4.5±0.5 | 4.5±0.7 | 5.0±0.0 | 4.5±0.8 |
| Patient 2 | 4.9±0.3 | 4.7±0.6 | 4.5±0.9 | 4.4±1.1 | 4.3±1.2 | 4.6±0.9 |
| Patient 3 | 4.7±0.8 | 4.4±1.0 | 3.5±1.5 | 4.3±0.9 | 5.0±0.0 | 4.4±1.1 |
| Patient 4 | 4.6±1.1 | 4.4±1.0 | 4.2±1.4 | 4.1±1.4 | 4.0±1.4 | 4.3±1.2 |
| Patient 5 | 4.7±0.7 | 4.2±0.9 | 4.3±0.8 | 4.1±1.2 | 5.0±0.0 | 4.5±0.9 |
| Patient 6 | 4.7±0.8 | 4.4±0.9 | 4.3±0.8 | 4.2±1.1 | 4.9±0.3 | 4.5±0.8 |
| Patient 7 | 4.7±0.6 | 4.4±1.0 | 4.0±1.1 | 4.5±0.8 | 5.0±0.0 | 4.5±0.9 |
| Patient 8 | 4.7±0.9 | 4.3±0.9 | 4.0±1.2 | 3.9±1.2 | 5.0±0.0 | 4.4±1.0 |
| Patient 9 | 4.5±0.9 | 4.2±0.9 | 4.2±0.9 | 4.2±0.9 | 4.2±0.9 | 4.3±0.9 |

**Supplementary Table 4.** Assessment of the simulated patients’ **mean arterial pressure** at each time period.

|  | T0 | T1 | T2 | T3 | T4 | Overall |
| --- | --- | --- | --- | --- | --- | --- |
| Patient 1 | 5.0±0.0 | 4.4±0.8 | 4.3±0.8 | 4.2±0.9 | 5.0±0.0 | 4.6±0.7 |
| Patient 2 | 5.0±0.0 | 4.9±0.4 | 4.7±0.7 | 4.5±1.2 | 4.5±1.1 | 4.7±0.8 |
| Patient 3 | 5.0±0.0 | 4.5±1.2 | 4.1±1.3 | 4.3±1.2 | 5.0±0.0 | 4.6±1.0 |
| Patient 4 | 4.7±0.8 | 3.9±1.1 | 3.9±1.2 | 3.7±1.2 | 3.6±1.4 | 3.9±1.2 |
| Patient 5 | 4.8±0.6 | 4.5±0.8 | 4.1±1.2 | 3.9±1.2 | 5.0±0.0 | 4.5±1.0 |
| Patient 6 | 4.9±0.3 | 4.4±1.0 | 3.9±1.4 | 3.7±1.5 | 5.0±0.0 | 4.4±1.1 |
| Patient 7 | 5.0±0.0 | 4.6±0.8 | 4.5±0.8 | 4.4±1.0 | 5.0±0.0 | 4.7±0.7 |
| Patient 8 | 4.8±0.8 | 4.0±1.1 | 3.6±1.3 | 4.1±0.9 | 5.0±0.0 | 4.3±1.1 |
| Patient 9 | 4.8±0.4 | 4.5±0.7 | 4.4±0.8 | 4.4±0.8 | 4.3±1.0 | 4.5±0.8 |

**Supplementary Table 5.** Assessment of the simulated patients’ **oxygen saturation (SpO_2_)** at each time period.

|  | T0 | T1 | T2 | T3 | T4 | Overall |
| --- | --- | --- | --- | --- | --- | --- |
| Patient 1 | 4.6±1.1 | 4.6±0.8 | 3.9±0.9 | 4.0±1.0 | 5.0±0.0 | 4.4±0.9 |
| Patient 2 | 4.7±0.7 | 4.8±0.4 | 4.5±1.1 | 4.3±1.2 | 4.1±1.1 | 4.5±0.9 |
| Patient 3 | 4.9±0.5 | 4.5±1.1 | 4.3±1.1 | 4.3±1.2 | 5.0±0.0 | 4.6±0.9 |
| Patient 4 | 4.5±1.1 | 4.5±0.7 | 4.6±0.8 | 4.5±1.1 | 4.6±1.1 | 4.6±0.9 |
| Patient 5 | 4.5±0.8 | 4.5±0.6 | 4.7±0.5 | 4.6±0.6 | 5.0±0.0 | 4.7±0.6 |
| Patient 6 | 4.7±0.7 | 4.5±0.8 | 4.5±0.8 | 4.5±0.6 | 5.0±0.0 | 4.7±0.7 |
| Patient 7 | 4.9±0.5 | 4.4±0.9 | 4.1±1.1 | 3.7±1.4 | 5.0±0.0 | 4.4±1.0 |
| Patient 8 | 4.8±0.6 | 4.3±1.3 | 4.3±1.3 | 4.5±1.1 | 5.0±0.0 | 4.6±1.0 |
| Patient 9 | 4.7±0.8 | 4.4±0.8 | 4.4±0.8 | 4.3±0.8 | 4.3±0.8 | 4.4±0.8 |

**Supplementary Table 6.** Assessment of the simulated patients’ **respiratory rate** at each time period.

|  | T0 | T1 | T2 | T3 | T4 | Overall |
| --- | --- | --- | --- | --- | --- | --- |
| Patient 1 | 4.9±0.3 | 4.3±0.9 | 4.2±0.9 | 4.2±0.9 | 5.0±0.0 | 4.5±0.8 |
| Patient 2 | 4.9±0.5 | 4.9±0.5 | 4.7±0.7 | 4.5±1.2 | 4.1±1.4 | 4.6±1.0 |
| Patient 3 | 5.0±0.0 | 4.5±1.2 | 4.1±1.2 | 3.1±1.3 | 5.0±0.0 | 4.3±1.2 |
| Patient 4 | 4.9±0.5 | 4.8±0.6 | 4.6±0.8 | 4.5±1.2 | 4.5±1.2 | 4.6±0.9 |
| Patient 5 | 4.9±0.5 | 4.7±0.7 | 4.5±0.9 | 3.9±1.0 | 5.0±0.0 | 4.6±0.8 |
| Patient 6 | 4.9±0.3 | 4.7±0.6 | 4.5±1.0 | 4.0±1.1 | 5.0±0.0 | 4.6±0.8 |
| Patient 7 | 4.9±0.3 | 4.1±1.2 | 3.7±1.1 | 4.5±0.9 | 5.0±0.0 | 4.5±0.9 |
| Patient 8 | 4.7±0.9 | 2.8±1.7 | 2.4±1.5 | 2.4±1.4 | 5.0±0.0 | 3.4±1.7 |
| Patient 9 | 5.0±0.0 | 4.8±0.6 | 4.8±0.6 | 4.7±0.7 | 4.7±0.7 | 4.8±0.6 |

**Supplementary Table 7.** Assessment of the simulated patients’ **Glasgow coma scale** at each time period.

|  | T0 | T1 | T2 | T3 | T4 | Overall |
| --- | --- | --- | --- | --- | --- | --- |
| Patient 1 | 4.7±0.6 | 3.9±1.0 | 4.6±0.7 | 4.9±0.3 | 5.0±0.0 | 4.6±0.7 |
| Patient 2 | 5.0±0.0 | 4.9±0.3 | 4.8±0.6 | 4.6±0.7 | 4.4±1.2 | 4.7±0.7 |
| Patient 3 | 4.8±0.4 | 4.2±1.1 | 3.2±1.3 | 4.9±0.4 | 4.9±0.3 | 4.4±1.0 |
| Patient 4 | 4.7±0.8 | 4.6±0.8 | 4.5±0.9 | 4.2±0.9 | 4.2±1.1 | 4.5±0.9 |
| Patient 5 | 5.0±0.0 | 4.5±0.7 | 4.2±1.1 | 3.3±1.4 | 5.0±0.0 | 4.4±1.0 |
| Patient 6 | 4.7±0.6 | 4.1±1.1 | 3.7±1.5 | 3.3±1.5 | 5.0±0.0 | 4.2±1.3 |
| Patient 7 | 4.9±0.5 | 4.2±1.1 | 3.1±1.3 | 4.5±1.1 | 4.9±0.3 | 4.3±1.1 |
| Patient 8 | 3.7±1.8 | 4.6±1.1 | 4.8±0.8 | 4.8±0.8 | 5.0±0.0 | 4.6±1.1 |
| Patient 9 | 4.5±1.1 | 4.5±1.2 | 4.5±1.2 | 4.5±1.2 | 4.5±1.2 | 4.5±1.2 |

**Supplementary Table 8.** Assessment of the simulated patients’ **ability to walk** at each time period.

|  | T0 | T1 | T2 | T3 | T4 | Overall |
| --- | --- | --- | --- | --- | --- | --- |
| Patient 1 | 4.5±1.1 | 4.3±1.1 | 3.1±1.3 | 3.0±1.3 | 4.7±0.9 | 3.9±1.3 |
| Patient 2 | 4.7±0.7 | 4.6±0.8 | 4.7±0.7 | 4.7±0.9 | 4.5±1.1 | 4.7±0.8 |
| Patient 3 | 4.7±0.6 | 4.4±1.1 | 3.5±1.2 | 3.5±1.3 | 4.9±0.5 | 4.2±1.2 |
| Patient 4 | 4.6±0.6 | 4.3±0.7 | 4.3±0.7 | 4.3±0.7 | 4.3±0.7 | 4.3±0.7 |
| Patient 5 | 4.7±0.6 | 4.5±0.7 | 4.1±0.7 | 4.1±0.7 | 5.0±0.0 | 4.5±0.7 |
| Patient 6 | 4.7±0.7 | 4.6±0.8 | 4.3±0.9 | 4.1±1.0 | 5.0±0.0 | 4.5±0.8 |
| Patient 7 | 4.9±0.5 | 4.7±0.7 | 4.7±0.7 | 4.5±0.8 | 5.0±0.0 | 4.7±0.6 |
| Patient 8 | 4.6±0.8 | 4.3±1.3 | 4.3±1.3 | 4.5±1.1 | 4.9±0.5 | 5.4±1.0 |
| Patient 9 | 4.7±0.7 | 3.5±1.3 | 3.5±1.3 | 3.5±1.3 | 3.5±1.3 | 3.8±1.3 |

**Supplementary Table 9.** Assessment of the simulated patients’ **alveolar volume** at each time period.

|  | T0 | T1 | T2 | T3 | T4 | Overall |
| --- | --- | --- | --- | --- | --- | --- |
| Patient 1 | 4.5±0.9 | 3.7±1.3 | 3.8±0.9 | 3.9±0.8 | 3.4±1.4 | 3.9±1.1 |
| Patient 2 | 4.9±0.3 | 4.8±0.6 | 4.7±0.8 | 4.7±0.8 | 4.6±1.1 | 4.7±0.7 |
| Patient 3 | 4.9±0.4 | 4.4±1.0 | 4.0±1.2 | 4.3±1.1 | 4.3±1.2 | 4.4±1.0 |
| Patient 4 | 4.7±0.6 | 4.2±0.9 | 4.0±1.1 | 3.9±1.2 | 3.9±1.3 | 4.1±1.1 |
| Patient 5 | 4.8±0.4 | 4.7±0.5 | 4.3±0.9 | 4.1±1.1 | 4.8±0.8 | 4.5±0.8 |
| Patient 6 | 4.9±0.4 | 4.7±0.5 | 4.2±0.9 | 4.0±1.2 | 4.7±0.8 | 4.5±0.9 |
| Patient 7 | 4.8±0.6 | 4.7±0.7 | 4.6±0.8 | 4.4±1.1 | 4.5±1.0 | 4.6±0.8 |
| Patient 8 | 4.7±0.9 | 4.2±1.1 | 4.1±1.0 | 4.5±0.7 | 4.7±1.0 | 4.4±1.0 |
| Patient 9 | 4.7±0.6 | 3.8±1.1 | 3.9±1.1 | 3.9±1.1 | 3.9±1.1 | 4.0±1.1 |

**Supplementary Table 10.** Assessment of the simulated patients’ **PaO_2_** at each time period.

|  | T0 | T1 | T2 | T3 | T4 | Overall |
| --- | --- | --- | --- | --- | --- | --- |
| Patient 1 | 4.5±0.9 | 4.1±1.0 | 2.5±1.2 | 2.9±1.2 | 3.7±1.4 | 3.5±1.4 |
| Patient 2 | 4.9±0.4 | 4.6±0.6 | 4.5±0.8 | 4.5±0.9 | 4.3±1.2 | 4.5±0.8 |
| Patient 3 | 4.8±0.4 | 4.3±1.2 | 3.2±1.1 | 3.5±1.2 | 4.1±1.3 | 4.0±1.2 |
| Patient 4 | 4.5±0.8 | 4.0±1.2 | 3.9±1.2 | 3.8±1.3 | 3.8±1.4 | 4.0±1.2 |
| Patient 5 | 4.7±0.6 | 4.5±0.9 | 4.1±1.1 | 4.0±1.1 | 4.5±0.7 | 4.4±0.9 |
| Patient 6 | 4.8±0.6 | 4.5±0.7 | 3.8±1.1 | 3.7±1.2 | 4.6±0.7 | 4.3±1.0 |
| Patient 7 | 4.7±0.6 | 4.5±1.0 | 4.3±1.0 | 3.9±1.2 | 4.6±0.9 | 4.4±1.0 |
| Patient 8 | 4.5±0.9 | 4.3±0.9 | 4.4±0.7 | 4.4±0.7 | 4.7±0.6 | 4.5±0.8 |
| Patient 9 | 4.9±0.3 | 2.7±1.4 | 2.7±1.4 | 2.9±1.4 | 2.9±1.4 | 3.2±1.5 |

**Supplementary Table 11.** Assessment of the simulated patients’ **PaCO_2_** at each time period.

|  | T0 | T1 | T2 | T3 | T4 | Overall |
| --- | --- | --- | --- | --- | --- | --- |
| Patient 1 | 4.3±1.1 | 4.3±1.0 | 4.2±0.8 | 3.9±0.9 | 4.1±1.0 | 4.2±0.9 |
| Patient 2 | 4.7±0.9 | 4.3±1.1 | 4.3±1.2 | 4.2±1.1 | 4.1±1.3 | 4.3±1.1 |
| Patient 3 | 4.6±0.9 | 4.3±1.3 | 4.2±1.3 | 4.0±1.4 | 4.5±0.9 | 4.3±1.2 |
| Patient 4 | 4.7±0.7 | 4.7±0.7 | 4.7±0.7 | 4.7±0.7 | 4.7±0.7 | 4.7±0.7 |
| Patient 5 | 4.7±0.6 | 4.7±0.6 | 4.7±0.6 | 4.7±0.6 | 4.7±0.7 | 4.7±0.6 |
| Patient 6 | 4.7±0.7 | 4.6±0.7 | 4.5±0.7 | 4.5±0.8 | 4.4±0.9 | 4.5±0.8 |
| Patient 7 | 4.9±0.5 | 4.7±0.6 | 4.7±0.6 | 4.7±0.6 | 4.6±0.9 | 4.7±0.6 |
| Patient 8 | 4.7±0.7 | 4.6±0.7 | 4.6±0.7 | 4.6±0.7 | 4.7±0.7 | 4.6±0.7 |
| Patient 9 | 4.7±0.8 | 4.7±0.8 | 4.7±0.8 | 4.7±0.8 | 4.7±0.8 | 4.7±0.8 |

**Supplementary Table 12.** Assessment of the simulated patients’ **blood volume** at each time period.

|  | T0 | T1 | T2 | T3 | T4 | Overall |
| --- | --- | --- | --- | --- | --- | --- |
| Patient 1 | 4.5±0.8 | 4.3±0.9 | 4.1±1.0 | 4.1±0.7 | 5.0±0.0 | 4.4±0.8 |
| Patient 2 | 4.7±0.8 | 4.6±0.7 | 4.6±0.9 | 4.4±1.1 | 4.3±1.2 | 4.5±0.9 |
| Patient 3 | 4.7±0.9 | 4.3±1.2 | 4.0±1.3 | 3.7±1.4 | 4.9±0.3 | 4.3±1.1 |
| Patient 4 | 4.8±0.6 | 4.5±0.9 | 4.3±1.0 | 4.3±1.0 | 4.3±1.0 | 4.4±0.9 |
| Patient 5 | 4.9±0.3 | 4.9±0.4 | 4.8±0.4 | 4.5±0.7 | 5.0±0.0 | 4.8±0.4 |
| Patient 6 | 4.7±0.6 | 4.7±0.6 | 4.5±0.8 | 4.2±1.3 | 5.0±0.0 | 4.6±0.8 |
| Patient 7 | 4.8±0.6 | 4.7±0.7 | 4.5±1.0 | 4.1±1.1 | 5.0±0.0 | 4.6±0.8 |
| Patient 8 | 4.6±0.7 | 4.1±1.1 | 3.9±1.3 | 3.9±1.2 | 4.9±0.5 | 4.3±1.1 |
| Patient 9 | 4.7±0.8 | 4.5±1.0 | 4.5±1.0 | 4.5±1.0 | 4.5±1.0 | 4.5±1.0 |

**Supplementary Table 13.** Assessment of the simulated patients’ **stroke volume** at each time period.

|  | T0 | T1 | T2 | T3 | T4 | Overall |
| --- | --- | --- | --- | --- | --- | --- |
| Patient 1 | 4.9±0.3 | 4.8±0.4 | 4.7±0.8 | 4.6±0.9 | 4.3±1.4 | 4.7±0.9 |
| Patient 2 | 4.9±0.5 | 4.7±0.7 | 4.7±0.7 | 4.7±0.7 | 4.6±1.1 | 4.7±0.8 |
| Patient 3 | 5.0±0.0 | 4.7±1.0 | 4.7±1.0 | 4.7±1.0 | 4.7±1.0 | 4.8±0.9 |
| Patient 4 | 4.9±0.5 | 4.9±0.5 | 4.9±0.5 | 4.8±0.8 | 4.8±0.8 | 4.8±0.6 |
| Patient 5 | 4.7±0.7 | 4.7±0.7 | 4.7±0.7 | 4.7±0.9 | 4.5±1.2 | 4.7±0.9 |
| Patient 6 | 4.9±.0.3 | 4.8±0.6 | 4.8±0.6 | 4.7±0.7 | 4.8±0.6 | 4.8±0.5 |
| Patient 7 | 4.9±0.5 | 4.7±0.6 | 4.5±0.8 | 4.4±1.0 | 4.4±1.2 | 4.6±0.9 |
| Patient 8 | 4.8±0.6 | 4.6±0.7 | 4.5±0.9 | 4.5±1.1 | 4.5±1.2 | 4.6±0.9 |
| Patient 9 | 5.0±0.0 | 4.8±0.6 | 4.8±0.6 | 4.8±0.6 | 4.8±0.6 | 4.8±0.5 |

**Supplementary Table 14.** Assessment of the simulated patients’ **intracranial pressure** at each time period.
